# Amelanocytic anorectal malignant melanoma—Case report

**DOI:** 10.1016/j.ijscr.2019.01.029

**Published:** 2019-01-31

**Authors:** Marta Serra, Teresa Santos, Margarida Martins, Leonor Sardo

**Affiliations:** Centro Hospitalar do Baixo Vouga – Av. Artur Ravara, 3810 – 501 Aveiro, Portugal

**Keywords:** AMM, anorectal malignant melanoma, APR, abdominoperineal resection, CT, computed tomography, ER, emergency room, HIV, human immunodeficiency virus, HPV, human papilomavirus, PET, positron emission tomography, RLL, right lower lobe, WLE, wide local excision, Anorectal malignant melanoma, Amelanocytic, Surgery, Wide local excision, Abdominal perineal resection, Case report

## Abstract

**Introduction:**

Anorectal malignant melanoma (AMM), is a rare and aggressive form of melanoma. Accounts for <2% of melanomas, and <2% of anal tumors. It is more frequent at the 6^th^–7^th^ decade, mostly in women. Surgery is the mainstay of treatment, but the extent is controversial.

**Case presentation:**

82-year-old male, presented with rectal mass in the Emergency Room (ER). After local excision, pathology observed that the mass corresponded to AMM. Despite being stage I (Local disease), a year later it presented with local recurrence and distant metastasis. The patient died 32 months after diagnosis.

**Conclusions:**

There is no optimal treatment, due to the lack of prospective studies. Wide local excision (WLE) has faster recovery, less post-operatory complications and similar survival rate compared to abdominal perineal resection (APR).

## Highlights

• Anorectal malignant melanoma, is a rare and aggressive form of melanoma.
• Very different from its cutaneous counterpart.
• There is no optimal treatment. Surgery is the mainstay of treatment, but the extent is controversial.
• Patient’s quality of life deserves attention when choosing the adequate surgical technic.

## 1 Introduction

AMM consists of a rare and aggressive form of melanoma, first reported by Moore et al. in 1857 [1]. Despite being the third most frequent location, after cutaneous and ocular, it accounts for less than 2% of all melanomas [2] and 24% of mucosal melanomas [3]. Corresponds to less than 2% of anal tumors [4].

It is most frequently diagnosed at an advanced age (6^th^-7^th^ decade) [5], due to its indolent course and nonspecific symptoms, resulting in large masses and disseminated disease at presentation. Its etiology is still unclear.

Surgical management is the treatment of choice, considering the low response to chemo and radiotherapy. The ideal surgical treatment is still controversial, especially in terms of extension: WLE *vs* APR.

AMM has poor prognosis. Recurrence and mortality are high, with a 5-year survival rate of less than 20% [1,6] and a median survival of less than 2 years [2,4].

We present a case of a patient with amelanocytic malignant melanoma and the evolution of the disease.

## 2 Case presentation

82-year-old Caucasian male, with history of prolapsed anal mass, was observed at the ER. Rectal examination revealed two large (2 cm and 1,8 cm), anterior, amelanocytic polyps, at 1 cm from the anal verge. The patient denied changes in bowel habits or other symptoms.

An elective trans-anal polypectomy was performed. Histopathology showed malignant melanoma: Mixed histology – Spindle and epithelioid cells; Thickness - 11 mm; 16 mitosis/mm2; positive margins. Immunohistochemistry showed positivity to PS100, CD117 and HMB45; and negativity to CAM 5.2, AE1/AE3, p63, CD34.

**Fig. 1:**
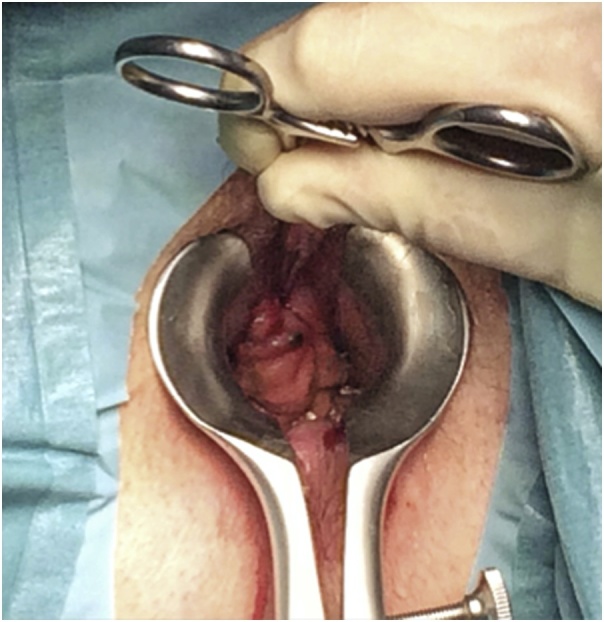
Second intervention – Melanocytic anal lesion.

After a multidisciplinary team discussion, and due to the patient’s old age, it was decided to propose a second conservative approach: Wide local excision (WLE) (Fig. 1). This time a melanocytic nodule was observed at the same localization of the previous mass. Pathologic examination showed AMM with pure spindle cell histology; 3 mm thick; margins free from disease. A thoracic, abdominal and pelvic CT scan was performed: No signs of distant metastasis.

The patient was sent to the National Cancer Center for follow up.

A year later the patient presented with loco regional recurrence - Anal mass and palpable inguinal nodes, without any constitutional symptoms; Underwent surgery once again: WLE and bilateral inguinal and iliac lymphadenectomy. Pathology observed anal recurrence (free margins <1 mm) and lymph node metastasis (15 out of 35 lymph nodes).

**Fig. 2:**
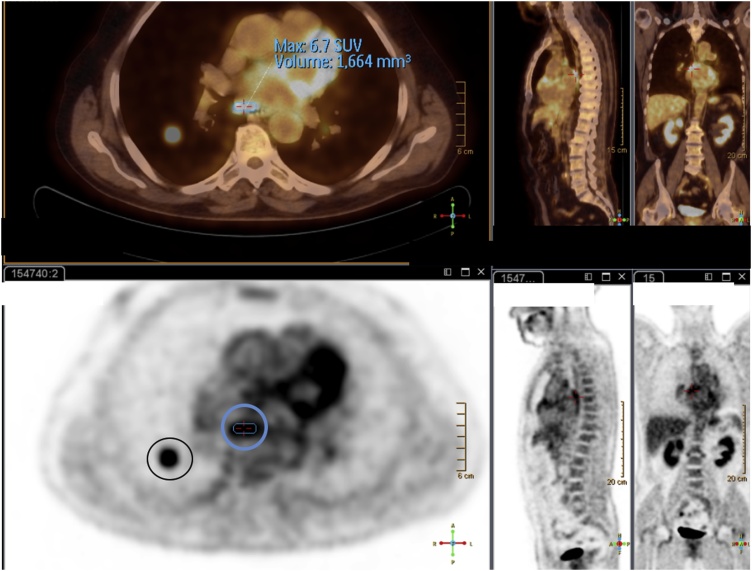
PET scan revealing RLL pulmonary (circled in black, lower image) and mediastinal nodal (circled in blue, upper and lower image) metastasis.

Three months later, the follow-up PET-CT scan revealed a pulmonary metastasis at the right lower lobe and mediastinal lymph node involvement (Fig. 2). Due to disseminated disease and overall status, the patient did not undergo radio or chemotherapy.

Follow-up was performed every three months with clinical examination and imaging (PET-CT or CT scan). The patient died 32 months after the diagnosis, due to disease progression.

## 3 Discussion

AMM is a particularly rare and aggressive form of melanoma comparing to the cutaneous form. As all melanomas, it is originated from melanocytes, derived from the embryologic neural crest cells. They can be found throughout the body, mainly in the epidermis. In the rectum, these cells are located at the anal transition and squamous zone. AMM tends to spread submucosally. About 20% of the lesions are amelanocytic, carrying worse prognosis, due to its invasive nature [1,2].

Lesions mostly grow in the anal canal, followed by dentate line and the rectum [9]. Considering the lymphatic drainage of the anorectal region, the most common areas of lymphatic metastasis are the inguinal, mesenteric, hypogastric and para-aortic lymph nodes. Distant metastasis can locate at the liver, lung, brain and bone [4,7]. For the staging of anorectal and vaginal malignant melanomas a clinical system is used, due to their rarity: Stage I – Local disease; Stage II – Regional nodal involvement; Stage III – Distant metastasis. On the other hand, the staging of head, neck and vulva mucosal melanomas is based on the American Joint Committee on Cancer TNM system [4,8].

The etiology is still controversial: UVB radiation is a well-known risk factor for cutaneous melanoma, but it is unclear its influence in the development of AMM. Some authors consider to be a higher incidence in patients infected with HPV and HIV, but it is also controversial [4].

In terms of incidence it is more frequent in the elders, with its peek at the 6^th^ and 7^th^ decades. It seems to be slightly more prevalent among women and Caucasian [4,[7], [8], [9]].

Due to its non-specific symptoms and rare incidence leading to low index of suspicion, up to 40% of the patients have metastatic disease at presentation [1,4]. Weinstock reported that by the time of diagnostic, 37% of patients had restricted local disease, 41% had regional spread and 22% had already distant metastasis [7]. Main complaints include bleeding (55% of patients), anal mass (34%) and pain (13%) [4], being mistaken for hemorrhoidal disease. Other complaints include pruritus, tenesmus and change in bowel habits. When metastasized, anemia, weight loss and fatigue, can be present [4].

An exhaustive diagnostic workup must be done: Colonoscopy to evaluate any synchronous lesions; Endoscopic Ultrasound to assess tumor thickness and local nodal status; Thoracic, abdominal and pelvic CT scan or PET-CT scan to determine nodal and distant metastasis. Histological markers like S-100, HMB-45 and Vimentin may be helpful at diagnostic elucidation [4,5].

Surgery is considered the pillar of treatment in AMM, however, it is still controversial the aggressiveness of the resection. In early, small studies APR was the standard procedure considering its superior control of local disease. Subsequent studies revealed that APR has no benefit in terms of overall survival when compared to WLE [1,2,4]. WLE has the advantages of having shorter operation time, less invasive procedure (No stoma, no urinary or sexual interference), faster recovery and minimal impact on bowel function [1,6].

Despite WLE having a higher local recurrence when compared to APR, the recurrence in AMM occurs, simultaneously, locally and with distant metastasis. Consequently, APR has no statistically significant advantage in terms of survival or recurrence rate [4]. On the other hand, WLE has better quality of life and less surgical complications. Nonetheless, WLE may not be possible in the presence of sphincter invasion, bleeding or obstruction.

The inexistence of randomized control trials remains due to the rarity of AMM. In the absence of oncological benefit between WLE and APR, the quality of life of these patients deserves attention [1].

In addition, prophylactic inguinal lymph node dissection does not contribute to increase the survival rate, including when clinically positive [1,4]. It is recommended to access the stage of metastatic disease, even though, it is not evident if nodal metastasis affect long-term outcome [1,4]. The value of sentinel node in AMM is still to be proven. Lymphadenectomy remains recommended, although, the prognosis does not improve and the surgical morbidity increases significantly [4].

No systemic therapy is yet considered standard of care in AMM. However, it appears to be beneficial the association of surgery and adjuvant chemotherapy, compared to surgery alone, regarding survival [4]. Medications used in adjuvant chemotherapy are cisplatin, vinblastine, dacarbazine, interferon B and IL-2. Darcabazine is the most used therapy, with partial response in 20% of patients after 4–6 months of treatment. Common toxicities are bone marrow suppression, nausea, vomiting and fatigue. Radiotherapy’s major benefit is observed in palliative cases [4]. One study has evidenced similar local control between APR and WLE with adjuvant radiotherapy [2].

AMM has a very poor prognosis, with a mean survival of 20 months after treatment [2,4]. As predicted, patients without lymph node metastasis have better outcome than those who do – Stage 2 and 3 patients have no long-term survival (5 year-survival rate of 20% *vs* 0%) [4]. With this in mind, APR is suggested for patients with tumors less than 2 mm deep and no lymph metastasis, for a higher opportunity of cure and survival.

There are a few histologic markers associated with prognosis: Tumor thickness/size, tumor necrosis and perineural invasion are associated with worse prognosis, the latter having the strongest association with poorer disease-specific and long-term survival. Tumor histologic type was involved with recurrence, but not with survival [3,4]: Pure epithelioid has smaller recurrence rate than pure spindle cell or mixed histology [3]. Tumor ulceration, lymphovascular invasion, presence of melanin and *in situ* melanoma had no interference with outcome [3,4]. Patients with symptoms had shorter recurrence-free and disease-specific survival. Sex, age, site of lesion and type of resection have no association with prognosis [3].

## 4 Conclusion

Anorectal malignant melanoma is a rare and aggressive tumor. The clinical, biological and molecular differences make the mucosal melanoma very distinct from its cutaneous counterpart. There is no optimal surgical treatment, due to the lack of prospective studies. However, WLE has faster recovery, less post-operatory complications and similar survival rate to APR.

This clinical case has been reported in line with the SCARE criteria [10]

## Conflicts of interest

Nothing to declare.

## Funding

Nothing to declare.

## Ethical approval

In my institution (Centro Hospitalar do Baixo Vouga, Aveiro, Portugal) the publication of clinical cases, especially those that do not expose the identity of the patient, does not require ethical approval.

## Consent

Considering there is not any signed consent from the deceased patient (C. P. V – Proc: 13006733), guardian or family, it is confirmed that exhaustive attempts have been made to contact the family and that the paper (“Amelocytic Anorectal Malignant Melanoma – Case Report”) has been sufficiently anonymised not to cause harm to the patient or their family.

## Author contribution

Marta Serra, Margarida Martins and Leonor Sardo performed the first two surgeries; Marta Serra wrote the manuscript. Teresa Santos contributed in data research. All authors read and approved the final manuscript.

## Registration of research studies

Not applicable – Case Report

## Guarantor

Marta Serra.

## Provenance and peer review

Not commissioned, externally peer-reviewed
